# Novel estrogen-responsive genes (ERGs) for the evaluation of estrogenic activity

**DOI:** 10.1371/journal.pone.0273164

**Published:** 2022-08-17

**Authors:** Kentaro Nishi, Wenqiang Fu, Ryoiti Kiyama

**Affiliations:** Department of Life Science, Faculty of Life Science, Kyushu Sangyo University Matsukadai, Higashi-ku, Fukuoka, Japan; Universita degli Studi di Salerno, ITALY

## Abstract

Estrogen action is mediated by various genes, including estrogen-responsive genes (ERGs). ERGs have been used as reporter-genes and markers for gene expression. Gene expression profiling using a set of ERGs has been used to examine statistically reliable transcriptomic assays such as DNA microarray assays and RNA sequencing (RNA-seq). However, the quality of ERGs has not been extensively examined. Here, we obtained a set of 300 ERGs that were newly identified by six sets of RNA-seq data from estrogen-treated and control human breast cancer MCF-7 cells. The ERGs exhibited statistical stability, which was based on the coefficient of variation (CV) analysis, correlation analysis, and examination of the functional association with estrogen action using database searches. A set of the top 30 genes based on CV ranking were further evaluated quantitatively by RT-PCR and qualitatively by a functional analysis using the GO and KEGG databases and by a mechanistic analysis to classify ERα/β-dependent or ER-independent types of transcriptional regulation. The 30 ERGs were characterized according to (1) the enzymes, such as metabolic enzymes, proteases, and protein kinases, (2) the genes with specific cell functions, such as cell-signaling mediators, tumor-suppressors, and the roles in breast cancer, (3) the association with transcriptional regulation, and (4) estrogen-responsiveness. Therefore, the ERGs identified here represent various cell functions and cell signaling pathways, including estrogen signaling, and thus, may be useful to evaluate estrogenic activity.

## Introduction

Estrogenic chemicals are found abundantly in natural and industrial materials, and they exhibit a variety of cellular and physiological activities [1]. Along with the increasing interest in the estrogenic activity of these chemicals and their beneficial applications, there is growing need to develop new technologies for their detection and characterization. Thus, a variety of assays have been developed, such as ligand-binding assays, reporter-gene assays, yeast two-hybrid assays, transcription assays, protein assays, cell assays, and animal tests, which are based on the mechanisms of estrogen action at the levels of molecules, cells, tissues, and the whole body [2]. Among the assays, gene expression profiling, such as the DNA microarray assay, is a technology used to detect the alterations of gene expression by monitoring the amount of mRNA or proteins of the estrogen-responsive genes (ERGs), which can detect the estrogenic activity of chemicals and mixtures of chemicals [3–5].

Searching for useful genes was challenging when the technological development was in its infancy. Recently, the development of DNA microarray technology for comprehensive searches of genes that were newly identified by the human genome project accelerated the search for ERGs, where tens to sometimes over a thousand ERGs were identified from more than ten thousand human genes and expressed sequence tags [6–11]. The identified ERGs have been examined for use in various fields such as food, clinical, pharmacological, and environmental applications [1]. For example, better understanding of the gene-regulation networks based on the ERGs is critical for the development of therapeutics for breast cancer [5]. Likewise, gene expression profiling based on ERGs has been used for environmental studies [12].

While the number of ERGs used for gene-expression profiling is important to reliably evaluate the estrogenic activity, the differences in the quality of ERGs due to various factors, such as their amounts in cells, the degrees of their responses, and their different cell functions, can cause unpredictable errors in the assay results. Other factors also need to be considered such as the cost, laboriousness, and the requirements of devices and equipment. The proportion of genes quantitatively showing differences in expression under different stimulations is roughly a few to a few tens of % of all human genes [13]. When considering statistical stability, the number of the genes that can be used to predict specific results, like clinical outcomes, should be no more than 1,000 and preferably less than 100 [13]. For example, MammaPrint, a US Food and Drug Administration-cleared molecular diagnostic test used for predicting the risk of breast cancer recurrence, contained 70 genes [14]. On the other hand, gene expression profiling has been used to predict estrogen activity, where a number of chemicals were examined by DNA microarray-based assays (reviewed by Kiyama & Zhu, 2014 [12]). A total of 120 ERGs, consisting of six functional groups of genes, were used to evaluate estrogenic activity, with comparable reliability to other estrogen assays [15]. However, methods for reliably identifying ERGs with statistical stability within satisfactory limits have not been reported, even though more information about genes and their functions has been obtained. Here, we determined the ERGs that have statistical stability based on RNA sequencing (RNA-seq) analysis and examined their usefulness by focusing on their reliability and applicability to predict the estrogen action of chemicals.

The recent progress in RNA-seq has enabled its wider use in applications such as food quality control, environmental materials, and medicine [16–20]. For estrogen actions, RNA-seq data obtained from estrogen-stimulated MCF-7 cells were compared with reverse transcription (RT)-PCR and DNA microarray data, suggesting mutually consistent or respectively complimentary gene expression profiles among them [21]. Furthermore, functional annotations of RNA-seq data with databases, such as the Gene Ontology (GO) and Kyoto Encyclopedia of Genes and Genomes (KEGG) databases, have been used to identify targets of antitumor agents against breast cancer such as diallyl trisulfide (a garlic metabolite) [22], 6-thioguanine [23], shikonin [24], tamoxifen [25], and chickpea isoflavones [26]. Based on RNA-seq analysis, novel mechanisms of cancer progression and metastasis involving non-coding RNAs [27, 28], enhancer RNAs [29], and circular RNAs [30, 31] were demonstrated. Functional regulatory networks examined by comparing chromatin immunoprecipitation-sequencing (ChIP-seq) data and time-course RNA-seq data revealed that transcription factor MYC plays an important role in connecting the networks between promoters and enhancers [32].

Here, we obtained a set of novel ERGs by RNA-seq that can be used for gene expression profiling of potential estrogenic chemicals and demonstrated their usefulness by examining their statistical reliability and their functional relevance.

## Materials and methods

### Materials

Human breast cancer MCF-7 cells were obtained from the Japanese Collection of Research Bioresources Cell Bank. Antibodies used for Western blotting were those against total Erk1/2 (T-Erk; #9102, Cell Signaling Technology, Ipswich, MA, USA), phospho-Erk1/2 (P-Erk; #9101), total Akt (T-Akt; #4691), and phospho-Akt (P-Akt; #4060).

### Sulforhodamine B (SRB) assay

MCF-7 cells were cultured in a phenol red-free RPMI 1640 medium (Gibco, Thermo Fisher Scientific, Waltham, MA, USA) supplemented with 10% fetal bovine serum (FBS) (Gibco, Thermo Fisher Scientific) and maintained at 37°C with 5% CO_2_ in an incubator (Thermo Fisher Scientific). The sulforhodamine B (SRB) assay was performed as described by Dong et al. [33, 34]. Before stimulation of MCF-7 cells with chemicals, the cells were cultured at a density of 1.5×10^4^ cells per well in RPMI 1640 containing 10% (v/v) dextran-coated charcoal-treated FBS (DCC-FBS) (Gibco, Thermo Fisher Scientific) for 3 days in 24-well plates. After treatment with 10 nM 17β-estradiol (E_2_; Sigma-Aldrich, St. Louis, MO, USA), 1 μM ICI 182,780 (ICI; Sigma-Aldrich), or 0.1% dimethyl sulfoxide (vehicle) (DMSO; FUJIFILM Wako Pure Chemical, Osaka, Japan) for 3 more days, MCF-7 cells were fixed with trichloroacetic acid (Sigma-Aldrich) at 4°C for 30 min and washed with ultrapure water. After washing, samples were stained with acetic acid containing 0.4% SRB (Sigma-Aldrich) at room temperature for 20 min and then washed with acetic acid. The bound protein was dissolved with 10 mM unbuffered Tris-base (pH = 10.5) at room temperature for 10 min and transferred into 96-well plates to measure the absorbance at 490 nm using the Modular-Designed Multimode Reader SH-9000 (Corona Electric, Ibaraki, Japan). Three independent assays were performed for each chemical, and the data were analyzed by *t*-test.

### Western blotting

Western blotting was performed as described by Dong et al. [33, 34]. MCF-7 cells at a density of 1.0×10^5^ cell per well were cultured for 2 days in DCC-FBS in 6-well plates and then one more day in serum-free medium. The cells were pretreated with ICI for 1 h and then treated with 10 nM E_2_ or vehicle (0.1% v/v DMSO). The total protein was extracted from the cells and examined by SDS-PAGE using e-PAGEL (ATTO, Tokyo, Japan) and electro-transferred onto nitrocellulose membranes (Millipore, Billerica, MA, USA) using a semi-dry transfer cell (Bio-Rad Laboratories, Benicia, CA, USA). The membranes were blocked with EzBlock BSA (ATTO) and incubated with the antibodies against signal proteins (T-Erk, P-Erk, T-Akt or P-Akt) overnight at 4°C after appropriate dilution (1:500 or 1:1000). The antibody-antigen complexes were detected with a horseradish peroxidase-coupled goat antibody against rabbit IgG (Cell Signaling Technology) after dilution (1:1000) and visualized using WSE-6100 LuminoGraph I (ATTO).

### RNA-seq analysis

MCF-7 cells at a density of 1.0×10^6^ cells per well were cultured in DCC-FBS for 3 days at 37°C in 5% CO_2_. The cells were treated with 10 nM E_2_, 1 μM ICI, or 0.1% DMSO (vehicle) for 2 more days. Total RNA was extracted using a RNeasy Mini-kit (QIAGEN, Venlo, the Netherlands). The DNA libraries for sequencing were constructed using the Truseq Stranded mRNA Library Prep (Illumina, San Diego, CA) and MGIEasy Universal Library Conversion Kit (MGI Tech, Shenzhen, PRC). Then, the libraries were subjected to sequencing using the DNBSEQ-G400 (MGISEQ-2000RS, MGI Tech) instrument, and the obtained reads were aligned using splitBarcode (https://github.com/MGI-tech-bioinformatics/splitBarcode). The read data were downsized to 20 M read pairs using seqkit v0.13.0 [35]. After the adopter sequences were cleaned with cutadapt ver.2.10 [36], the read data were mapped with HISAT v2.2.1 [37]. The value of FPKMs (reads per kilobase of transcript per million reads mapped) for each gene was estimated with Cuffdiff v2.2.1 [38], and the gene expression was analyzed as described previously [15]. After RNA-seq was repeated 6 times for E_2_, the standard deviation (SD) and the average (Av) for the 203 genes, which includes ERGs and control genes [15], were determined and used for calculating the log_2_ of FPKMs. These genes were evaluated based on the absolute value of SD/Av and used in the correlation analysis with the correlation coefficient (*R* value) between one of the assays (E_2_-1) and each of the other five assays (E_2_-2~ E_2_-6).

### *Real-time* RT-PCR

The FPKM values obtained with RNA-seq were log_2_-transformed and a total of 300 genes were selected based on the log_2_ value (gene expression ratio). Among the 300 genes, 30 genes were selected by ranking the SD/Av values and further analyzed by *real-time* RT-PCR [8]. *Real-time* RT-PCR was conducted with the CFX Connect *real-time* PCR detection system using the iTaq Universal SYBR Green One-Step Kit (Bio-Rad). The conditions for *real-time* RT-PCR were as follows: reverse transcription for cDNA at 42°C for 10 min and denaturation at 95°C for 1 min, followed by 46 cycles of denaturation at 94°C for 10 sec, annealing at 57°C for 30 sec, and extension at 72°C for 20 sec. After PCR, a melting curve was constructed by increasing the temperature from 65 to 95°C to confirm the validity of the reaction. *Real-time* RT-PCR was repeated 3 times for each gene and the Av and SD were calculated using CFX Maestro Software (Bio-Rad). The nucleotide sequences of PCR primers (S1 Table) were obtained from the references for *EGR3* [8], *LOXL2* [39], and *SYNE1* [40], or otherwise by web-based calculation.

### Data analysis for gene expression

The FPKM values obtained by RNA-seq were log_2_-transformed and *p*-values were calculated. The genes showing statistically significant (*p* < 0.05) up-regulation or down-regulation, or those without significant changes, were visualized by Volcano plotting [26], where log_2_-transformed expressional changes (X-axis) and -log_10_-transformed *p*-values or SD/Av values (Y-axis) are indicated. The pathway analysis was performed with WebGestalt software (WEB-based Gene SeT AnaLysis Toolkit) (www.webgestalt.org/; [41]) using the gene sets showing statistical significance (*p* < 0.05) from the GO (www.geneontology.org/) and KEGG (www.genome.jp/kegg/) databases. False discovery rates (FDRs: *Q*-values) were estimated using the Benjamini-Hochberg method, and the top ten categories of FDR < 0.05 were visualized.

## Results

### Gene expression profiling based on RNA-seq

**Fig 1:**
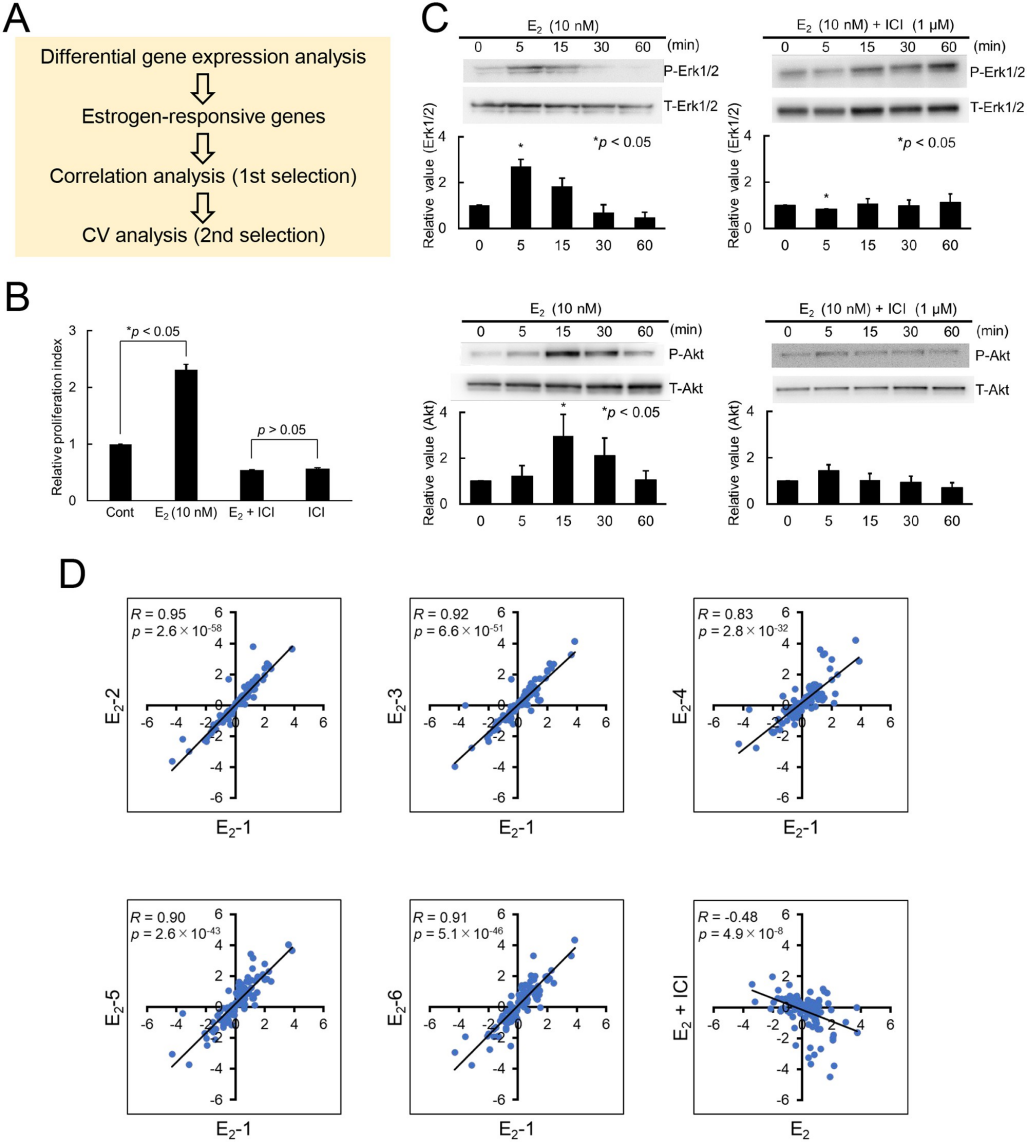
Evaluation of estrogenic activity by gene expression profiling. (A) A strategy adopted here. (B) Cell proliferation assay. MCF-7 cells treated with E_2_ (10 nM), E_2_ (10 nM) + ICI (1 μM), or vehicle (0.1% DMSO) were subjected to cell proliferation assays with SRB. The relative proliferation index for MCF-7 cells treated with E_2_ (10 nM), E_2_ (10 nM) + ICI (1 μM), or ICI (1 μM) alone are shown: *p < 0.05, vs. control (0.1% DMSO). (C) Western blotting. MCF-7 cells were treated with E_2_ (10 nM), E_2_ (10 nM) + ICI (1 μM), or vehicle (0.1% DMSO) for indicated times and cell extracts were subjected to Western blotting for the evaluation of phosphorylated proteins (P-Erk1/2 and P-Akt) (upper images) and total proteins (T-Erk1/2 and P-Akt) (lower images). (D) Correlation analysis of RNA-seq data. The gene expression profiles for MCF-7 cells treated with E_2_ obtained by RNA-seq were compared using a set of the 120 ERGs, and the results are visualized in scatter-plot graphs. The vertical and horizontal axes indicate log_2_-values of FPKMs. *R*- and *p*-values were calculated for each graph based on linear regression between two profiles.

Gene expression profiling is a method to evaluate the estrogenic activity of chemicals, and a variety of assays have been developed based on transcriptomic and proteomic technologies [1]. Here, we screened ERGs for evaluating estrogenic activity by transcriptomic assays such as RNA-seq and RT-PCR. To obtain a set of ERGs, we adopted a two-step process involving a correlation analysis and a coefficient of variation (CV) analysis (Fig 1A). MCF-7 cells were used as a cell system for the evaluation, which was performed first by two assays, a cell proliferation assay (SRB assay; Fig 1B) and protein assay (Western blotting to detect the phosphorylation of two marker proteins, Erk1/2 and Akt; Fig 1C), which have frequently been used to evaluate estrogenic activity [42–44]. The cells showed the response to estrogen (17β-estradiol; E_2_), and the response was inhibited by treatment with an ER antagonist ICI in both assays in the same way as previously reported [15], suggesting that the cells responded to E_2_ through the ER. Then, we performed the RNA-seq analysis to evaluate the cell response at the transcription level (Fig 1D). A total of six sets of gene expression profiles for MCF-7 cells treated with E_2_ were obtained by RNA-seq, where a set of the 120 ERGs, which were previously used in DNA microarray assays [15], were used to compare the profiles (first five panels in Fig 1D). The six combinations of profiles showed *R*-values of 0.83 to 0.95, indicating strong and statistically significant correlations among the profiles (see the data for all combinations in S2 Table). In contrast, there was no correlation between the profiles for the E_2_ treatment and E_2_+ICI treatment (the last panel in Fig 1D), suggesting the response to be mediated by the ER. Therefore, the ERGs that we obtained using gene expression profiling methods and by RNA-seq techniques can be used as markers for the evaluation of estrogenic chemicals.

### Evaluation of RNA-seq-based gene expression profiling

**Fig 2:**
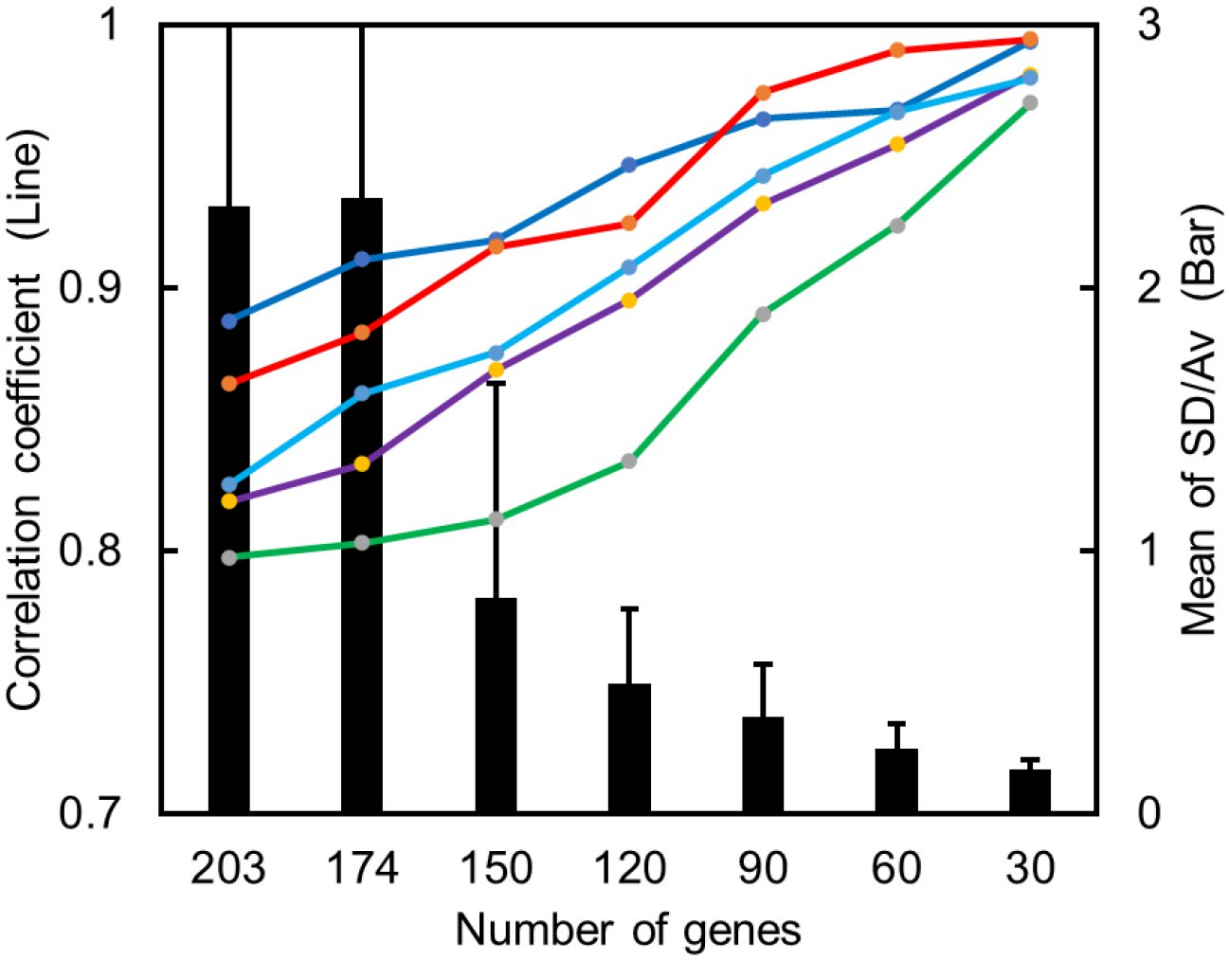
Statistical evaluation of ERGs. The RNA-seq for E_2_ was repeated 6 times and gene expression profiles were obtained (E_2_-1 to E_2_-6). The correlation coefficients (*R*-values) between one profile (E_2_-1) and the others (E_2_-2 to E_2_-6), giving five sets of data, were calculated for respective numbers of gene sets. The *R*-values of the total 203 genes, or the indicated numbers (174, 150, 120, 90, 60, or 30) of genes taken from the top of the list in the order of the genes ranked by the values of SD/Av indicating the statistical stability, are shown for the five sets of data in a line graph. The mean of SD/Av for respective numbers of genes are shown in a bar graph; SD/Av for 203 genes is 2.31 ± 6.24 and that for 174 genes is 2.34 ± 6.6.

Next, we examined the quality of the obtained ERGs by CV analysis (Fig 2). Among the six sets of ERGs obtained by RNA-seq (Fig 1D), the sets of 203 ERGs that were used to evaluate estrogenic activity by DNA microarray assays [15] were again examined by CV analysis (indicated by the bar graph in Fig 2), where the 203 genes included expression standards and 174 original ERGs, and the sets of 150, 120, 90, 60, or 30 genes were selected from the top of the list of genes ranked by CV values (mean of SD/Av). The top-ranked ERGs exhibited low CV values, 0.17 +/- 0.04 for the top 30 ERGs, indicating that gene expression profiling by RNA-seq can provide mean CV values that are lower than those by DNA microarray assays (mean CV value of 0.22; [15]). Furthermore, the correlation coefficients (*R*-values) for the top 30 ERGs in the five data sets obtained by RNA-seq were 0.97 to 0.99, which are also more reliable than those with nine data sets obtained by DNA microarray assays (0.93 to 0.97; [15]), confirming that the statistical stability of data is greater in RNA-seq than in DNA microarray assays.

### Selection of new ERGs for gene expression profiling

**Fig 3:**
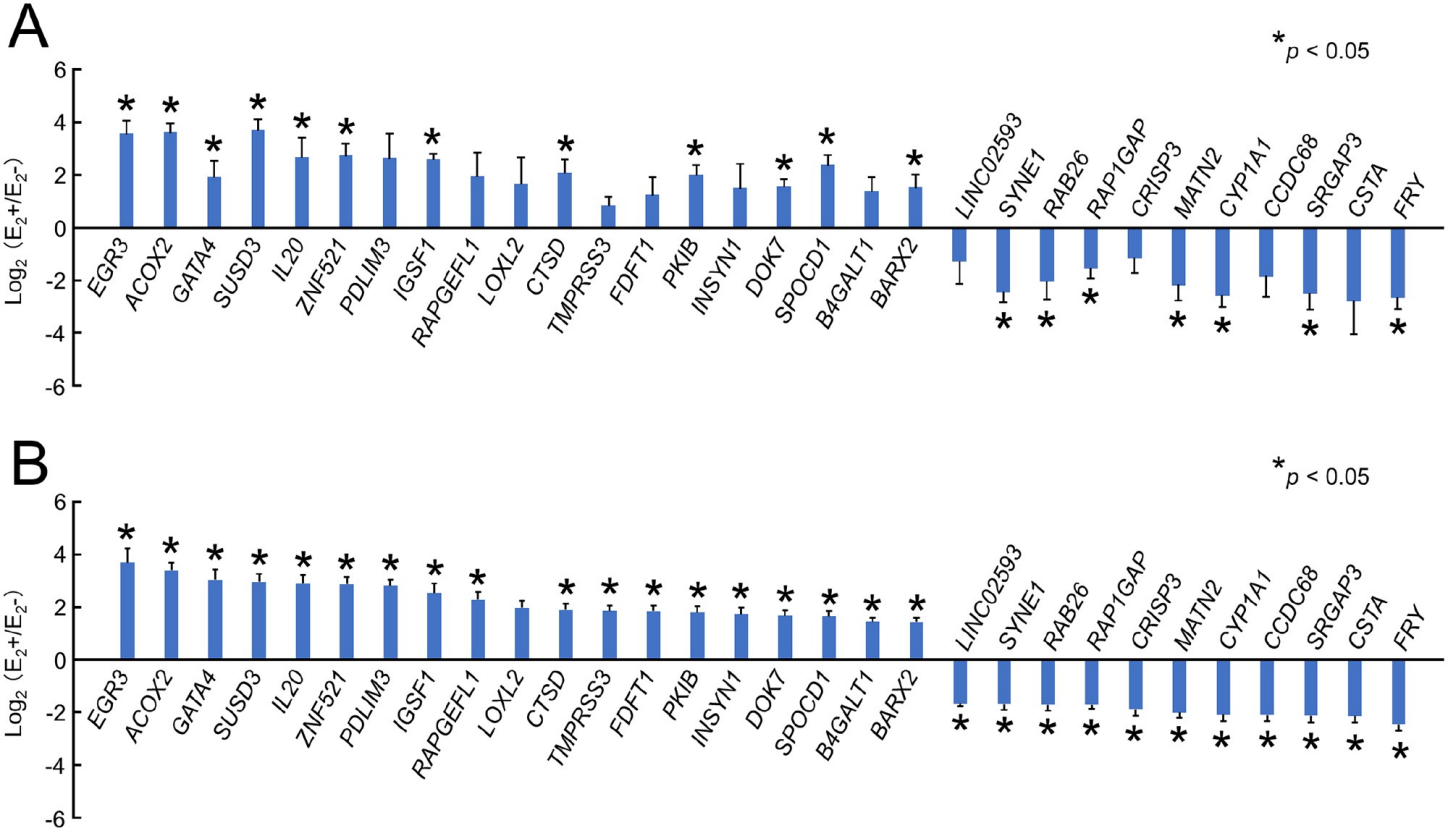
Transcriptional analysis of ERGs. (A) *Real-time* RT-PCR analysis for 30 ERGs. The 30 ERGs (listed in Table 1) that were selected by RNA-seq analysis in Fig 2 were analyzed again by *real-time* RT-PCR. The expression level of each gene was normalized by that of β-actin and indicated in a bar graph, where the bars show the mean ± SD (n = 3) of log_2_-transformed ratios of E_2_+ data and E_2_- data (E_2_+/E_2_-). (B) RNA-seq analysis for 30 ERGs. The values of FPKMs (n = 6) obtained by RNA-seq were log_2_-transformed and indicated in a bar graph, where the bars show the mean ± SD (n = 6) of the ratios, as shown in panel A. The top 30 genes selected according to SD/Av and expression ratios have stable gene expression. *p < 0.05: between E_2_ and the control (0.1% DMSO).

We selected new ERGs for gene expression profiling based on the approach shown in the previous section. A total of 300 ERGs were selected from the ranks created by CV analysis, from all human genes available for RNA-seq (a total of approx. 26,000 genes), which consist of 150 up-regulated and 150 down-regulated genes (see S3 Table for the complete list). Among the 300 ERGs, the top 30 ERGs (Table 1) were examined by *real-time* RT-PCR (Fig 3A), and the data were compared with those obtained by RNA-seq (Fig 3B). Both datasets were identical, except for minor differences due to slight variation in the locations of the transcripts that were detected by these assays. Notably, the statistical significance levels are lower for some genes, probably due to the lack of expression levels in the statistical consideration.

**Table 1:** List of 30 ERGs.

Rank	Gene symbol	CV	Rank	Gene symbol	CV
1	*LINC02593*	0.058	16	*DOK7*	0.125
2	*PDLIM3*	0.083	17	*CYP1A1*	0.125
3	*RAP1GAP*	0.090	18	*LOXL2*	0.126
4	*ACOX2*	0.091	19	*RAPGEFL1*	0.126
5	*B4GALT1*	0.094	20	*PKIB*	0.126
6	*TMPRSS3*	0.096	21	*CCDC68*	0.126
7	*MATN2*	0.098	22	*GATA4*	0.128
8	*SUSD3*	0.099	23	*SPOCD1*	0.129
9	*FDFT1*	0.099	24	*CRISP3*	0.134
10	*ZNF521*	0.099	25	*IGSF1*	0.134
11	*FRY*	0.104	26	*RAB26*	0.136
12	*BARX2*	0.107	27	*EGR3*	0.139
13	*IL20*	0.108	28	*SRGAP3*	0.139
14	*CTSD*	0.118	29	*SYNE1*	0.143
15	*CSTA*	0.124	30	*INSYN1*	0.145

The top 30 ERGs among the 300 genes stably responding to E_2_ are listed.

### Functional analysis of ERGs

**Fig 4:**
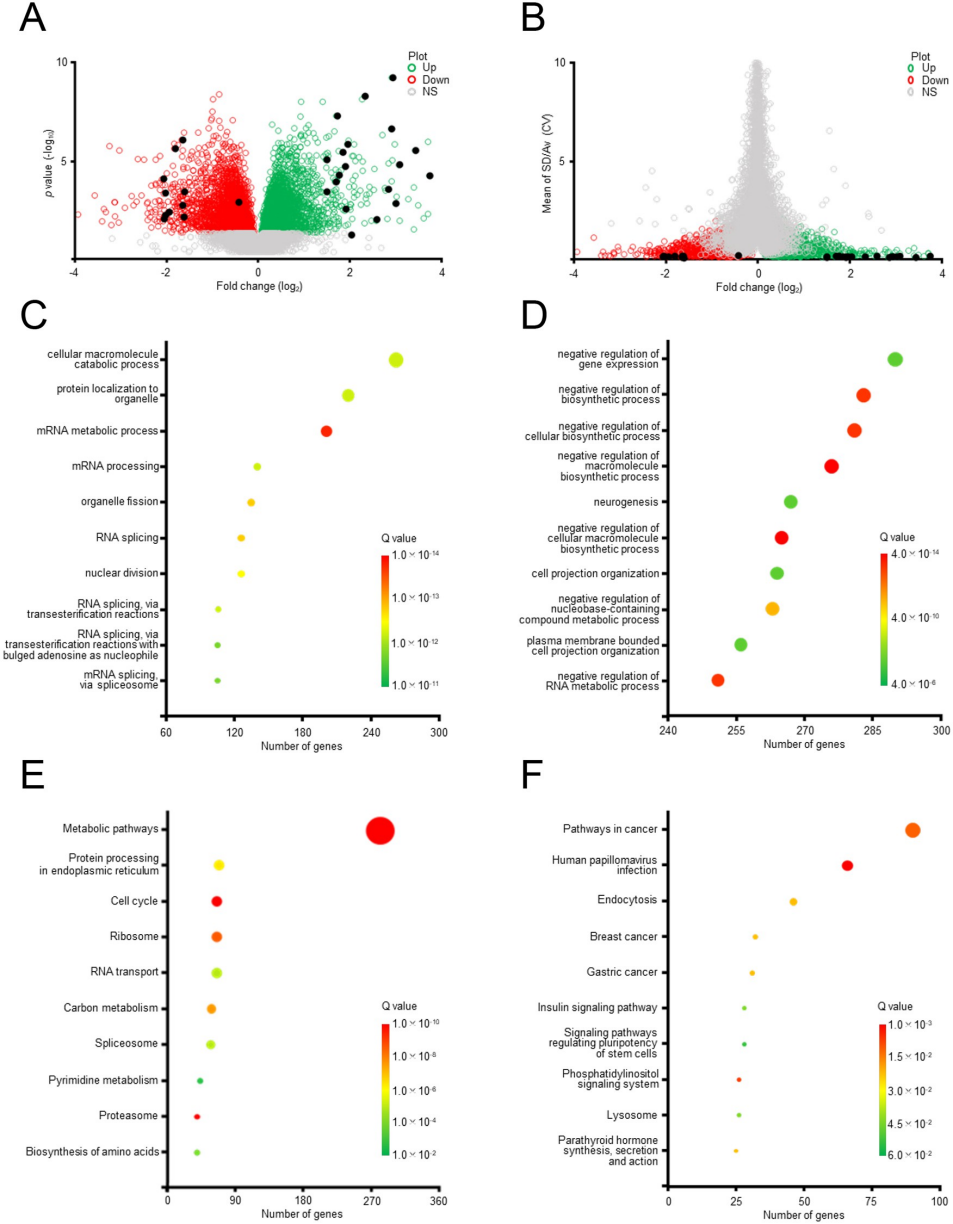
Signaling pathways associated with ERGs. (A) Volcano plots for ERGs in MCF-7 cells. The genes showing significant (*p* < 0.05) up-regulation (red circles) or down-regulation (green circles) are indicated, while those that are not significant are shown by gray circles. The novel 30 ERGs are shown by black dots. The graph shows log_2_-transformed fold-changes (X-axis) and -log_10_ of *p*-values (Y-axis). (B) Volcano plots for the 30 genes that show stable expression in response to E_2_. The graph shows log_2_-transformed fold-changes (X-axis) and mean of SD/Av values (Y-axis). (C, D) GO analysis for the biological processes (BPs). The top 10 significantly affected (*Q* < 0.05) BPs for the ERGs that exhibit up-regulation (panel C) or down-regulation (panel D) are shown. The number of genes in each group is visualized by the size of the circles, and the *Q*-values of the group by color. (E, F) KEGG analysis for metabolic pathways. The top 10 significantly affected (*Q* < 0.05) metabolic pathways for the ERGs that exhibit up-regulation (panel C) or down-regulation (panel D) are shown.

The newly identified ERGs were examined by signaling pathway analysis using the GO and KEGG databases (Fig 4). The characteristics of ERGs were analyzed first by the statistical evaluation of the relationship of the significance (*p*-values; Fig 4A) or stability (CV values; Fig 4B) of expressional alterations with the degrees of the alterations (log_2_-transformed fold changes) and then by the functional searches in the GO (Fig 4C and 4D) and KEGG (Fig 4E and 4F) databases. We confirmed that 30 ERGs belong to the groups of genes showing statistically significant alterations of their expression due to the treatment with estrogen (red or green circles in Fig 4A and 4B). From the database searches, significant levels of association were found between the genes of significant alterations of their expression and specific cell functions and pathways such as the cellular macromolecule catabolic process (a 262-gene group showing up-regulation), protein localization to organelles (a 220-gene group showing up-regulation), mRNA metabolic process (a 201-gene group showing up-regulation), negative regulation of gene expression (a 290-gene group showing down-regulation), negative regulation of biosynthetic process (a 283-gene group showing down-regulation), negative regulation of cellular biosynthetic process (a 281-gene group showing down-regulation), and negative regulation of macromolecule biosynthetic process (a 276-gene group showing down-regulation) in the GO database (Fig 4C and 4D; S4 Table), and metabolic pathways (a 279-gene group showing up-regulation), protein processing in endoplasmic reticulum (a 58-gene group showing up-regulation), cell cycle (a 55-gene group showing up-regulation), pathways in cancer (a 90-gene group showing down-regulation), human papillomavirus infection (a 66-gene group showing down-regulation), endocytosis (a 46-gene group showing down-regulation), and breast cancer (a 32-gene group showing down-regulation) in the KEGG database (Fig 4E and 4F; S4 Table). Furthermore, significant numbers of the 30 and 300 ERGs that were characterized in Fig 2 were included in the groups of genes found by the database searches (S4 Table), suggesting the association of these ERGs with specific cell functions.

### ER subtype-specific interaction of ERGs

**Fig 5:**
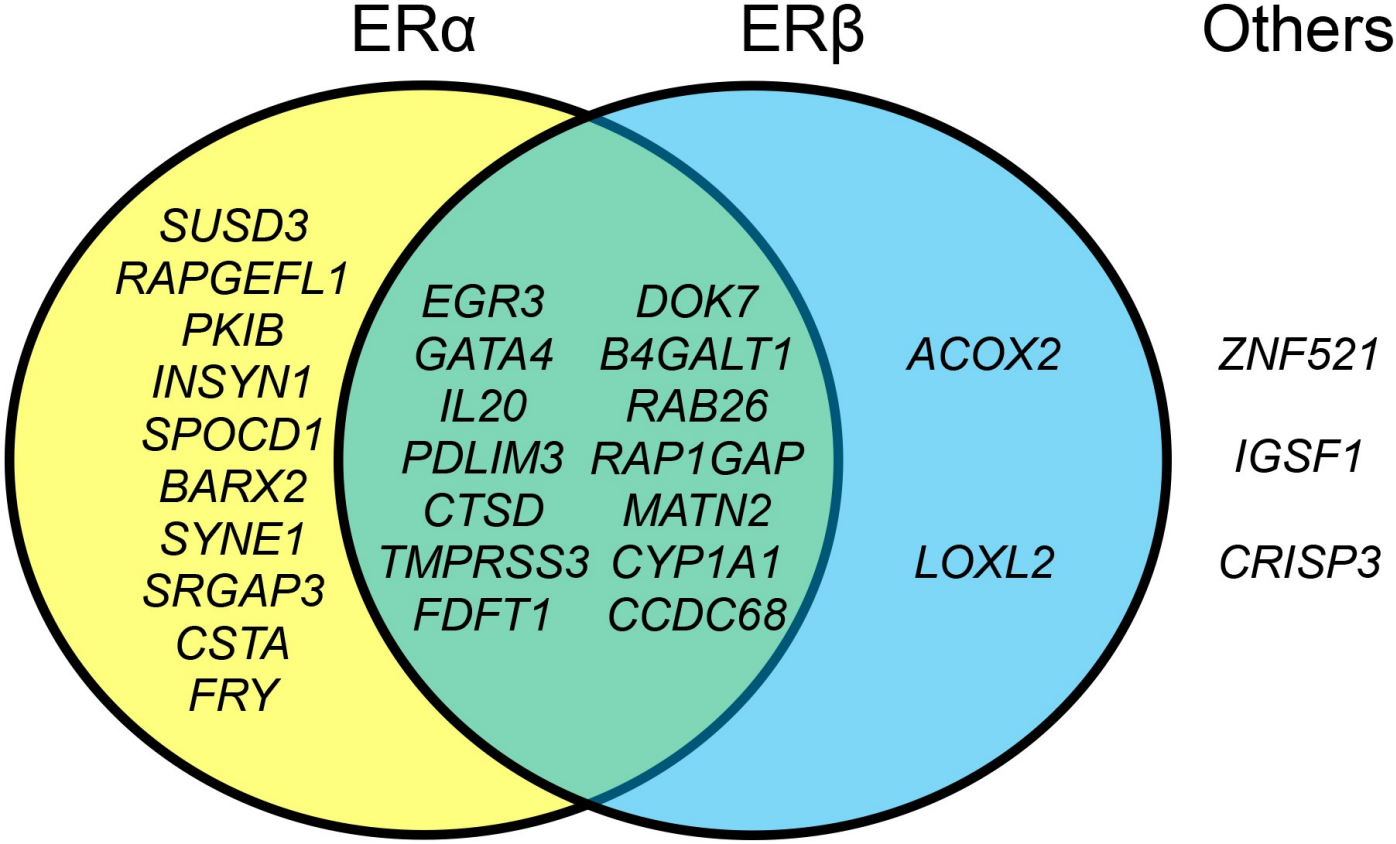
ER subtype-specific interaction of ERGs.

Since ERGs are likely controlled by ERs through the interaction at their promoter and enhancer regions, we examined the ChIP-seq data that are available in the Gene Expression Omnibus database to determine whether the regions containing these ERGs were identified as ER subtype-specific binding sites (Table 2). The results indicate that 29 ERGs (except *LINC02593*) were classified into four groups; ERα-specific (*SUSD3*, *RAPGEFL1*, *PKIB*, *INSYN1*, *SPOCD1*, *BARX2*, *SYNE1*, *SRGAP3*, *CSTA*, and *FRY*), ERβ-specific (*ACOX2* and *LOXL2*), ERα/β-specific (*EGR3*, *GATA4*, *IL20*, *PDLIM3*, *CTSD*, *TMPRSS3*, *FDFT1*, *DOK7*, *B4GALT1*, *RAB26*, *RAP1GAP*, *MATN2*, *CYP1A1*, and *CCDC68*), and other types (*ZNF521*, *IGSF1*, and *CRISP3*) (Fig 5).

**Table 2:** ER subtype-specific interaction of ERGs.

Gene No.	Gene symbol	ERα^a^		Erα binding^c^	ERβ^a^	Erβ binding^e^	Receptor subtype
		E_2_- *Q*-value^b^ (n = 3)	E_2_+ *Q*-value^b^ (n = 3)		E_2_+ Average of S/N ratio^d^ (n = 2)		
1	*EGR3*	0	279	+	1.1	+	ERα/β
2	*ACOX2*	0	0	-	1.4	+	ERβ
3	*GATA4*	133	2133	+	1.2	+	ERα/β
4	*SUSD3*	449	3026	+	0.7	-	ERα
5	*IL20*	1307	3091	+	1.1	+	ERα/β
6	*ZNF521*	0	0	-	0.9	-	-
7	*PDLIM3*	0	72	+	1.1	+	ERα/β
8	*IGSF1*	0	0	-	1.0	-	-
9	*RAPGEFL1*	764	4529	+	0.0	-	ERα
10	*LOXL2*	0	0	-	8.2	+	ERβ
11	*CTSD*	234	1683	+	4.4	+	ERα/β
12	*TMPRSS3*	263	2761	+	3.2	+	ERα/β
13	*FDFT1*	1121	3753	+	1.2	+	ERα/β
14	*PKIB*	304	1887	+	0.8	-	ERα
15	*INSYN1*	200	3575	+	1.0	-	ERα
16	*DOK7*	676	2677	+	32.7	+	ERα/β
17	*SPOCD1*	0	31	+	1.0	-	ERα
18	*B4GALT1*	145	1105	+	18.9	+	ERα/β
19	*BARX2*	89	465	+	1.0	-	ERα
21	*SYNE1*	0	395	+	1.0	-	ERα
22	*RAB26*	118	991	+	4.8	+	ERα/β
23	*RAP1GAP*	45	133	+	2.1	+	ERα/β
24	*CRISP3*	0	0	-	0.8	-	-
25	*MATN2*	0	200	+	2.5	+	ERα/β
26	*CYP1A1*	1203	2489	+	12.3	+	ERα/β
27	*CCDC68*	0	1490	+	1.2	+	ERα/β
28	*SRGAP3*	125	1290	+	0.9	-	ERα
29	*CSTA*	124	840	+	1.0	-	ERα
30	*FRY*	0	117	+	0.7	-	ERα

Gene No. is based on the order of the data from *real-time* RT-PCR (Fig 3). Gene No. 20 (*LINC02593*) is not listed here because it is a long non-coding RNA and inappropriate for the analysis.

^a^ The original data analyzed in this table are available in the NCBI’s Gene Expression Omnibus with accession numbers GSE117569 (for ERα; [135]) and GSE149979 (for ERβ; [151]).

^b^ The *Q*-value was determined using the ChIP-Atlas database (https://chip-atlas.org/; [152]), where each gene was analyzed for the presence of peaks within ± 10 kb from the transcription start site (TSS).

^c^ ERα binding is positive (+) when the *Q*-value of E_2_+ is bigger than that of E_2_-.

^d^ The integrative genomics viewer (IGV) was used to visualize the results of ChIP-seq, where each gene was analyzed for the presence of peaks within ± 10 kb from the TSS. Then, the S/N ratio was calculated, where the average of E_2_+/ERβ ChIP-seq values, rep 1 and 2, was used as S, and the E_2_+/input value was used as N.

^e^ ERβ binding is positive (+) when the S/N ratio is bigger than 1.0.

## Discussion

### ERGs for the evaluation of estrogenic activity

Estrogen is a hormone that regulates the female reproductive system and the physiological, neurological, and behavioral activities of females and, to some extent, males. Estrogen action is regulated and mediated by various genes and their protein products, including ERGs. Thus, ERGs can represent various estrogenic cell functions, and therefore, have been used as markers for estrogenic activity (reviewed in Kiyama & Zhu, 2014 [4]). Previously, specific ERGs were used as markers for gene expression or used as reporter-genes, which were later replaced by a set of ERGs in more statistically reliable transcriptomic assays such as DNA microarray assays and RNA-seq. A small number of ERGs, tens to hundreds, were enough for statistical stability, and including the genes without any response would increase the noise level. However, the quality of ERGs has not been extensively explored. Here, we found a set of 300 ERGs newly identified by six sets of RNA-seq data obtained from E_2_-treated and control MCF-7 cells (Figs 1 and 2). A set of the 30 genes with best statistical stability were further evaluated quantitatively by RT-PCR (Fig 3), and then qualitatively by a functional analysis using the GO and KEGG databases (Fig 4) and by a mechanistic analysis using ChIP-seq data (Fig 5). Since the 30 ERGs identified here exhibited alterations in gene expression at significant levels, they are associated with various cell functions and cell signaling pathways. How the genes are functionally associated with estrogen action and how they respond to estrogen are the questions that need to be examined further.

### Functional significance of ERGs

The 30 ERGs analyzed here can be classified into several functional categories. Firstly, the genes that encode enzymes, including enzyme activators and inhibitors, such as metabolic enzymes, proteases, and protein kinases. The genes encoding metabolic enzymes are: *ACOX2* encoding branched-chain acyl-CoA oxidase, a peroxisomal enzyme involved in the metabolism of branched-chain fatty acids and bile acid intermediates [45]; *B4GALT1* encoding β-1,4-galactosyltransferase 1, which transfers galactose in a β-1,4-linkage to *N*-acetylglucosamine, glucose, and xylose to form a lactosamine or lactose [46]; *CYP1A1* encoding a cytochrome P450 family 1A enzyme with monooxygenase or oxidoreductase activity [47, 48]; *FDFT1* encoding farnesyl-diphosphate farnesyltransferase 1, or squalene synthase, which catalyzes the conversion of *trans*-farnesyl-diphosphate to squalene, the first specific step in cholesterol biosynthesis [49]; *LOXL2* encoding a lysyl oxidase or protein lysin 6-oxidase, which catalyzes cross-linking collagen and elastin in the extracellular matrix [50–52]. The genes associated with proteolytic activity are as follows: *CRISP3* encoding a protein with homology to plant defense proteins with lytic activity against infectious pathogens [53]; *CSTA* encoding cystatin A, a cysteine proteinase inhibitor that belongs to a large family of the cystatin superfamily [54]; *CTSD* encoding cathepsin D, a lysosomal aspartic protease with many additional functions such as those in cell proliferation, invasion, metastasis, and angiogenesis of cancers [55]; *TMPRSS3* encoding a Type II transmembrane serine protease, a membrane-bound proteolytic enzyme that is associated with biological processes such as poor prognosis in patients with breast cancer [56, 57]. The genes associated with protein kinase activity and related cellular signaling are: *PKIB* encoding a protein acting as a competitive inhibitor of the cAMP-dependent protein kinase, which plays important roles in cell signaling in diseases such as the down-regulation of Akt signaling in irritable bowel syndrome [58], preeclampsia [59], and cancers [60–62]; *PKIB* encoding a competitive inhibitor of cAMP-dependent protein kinase involved in breast cancer development by enhancing ERα action [63]; *RAB26* encoding a RAB-family small GTPase that regulates intercellular vesicle trafficking, including exocytosis, endocytosis, and recycling [64]; *RAP1GAP* encoding a GTPase-activating protein (GAP) that promotes the hydrolysis of GTP in RAP1-GTP and inactivates RAP1, which plays important roles in the regulation of cell adhesion and migration and in the progression and metastasis of several types of cancer [65]; *RAPGEFL1* encoding a protein with predicted guanyl-nucleotide exchange factor (GEF) activity, which promotes RAP1 to load GTP and acquire the active GTP-bound status [66]; *RAPGEFL1*, a gene found in several types of cancer, acting as an estrogen-regulated gene in breast cancer cells [67]; *SRGAP3* encoding a protein highly expressed in the brain, with structural characteristics of BAR/Rac1 GAP/SH3 domains and considered to be a tumor suppressor in breast cancer [68].

Secondly, the genes that are related to specific cell functions and cancer are included in the 30 ERGs. CRISP3 protein has been known for its androgen-responsive gene expression [69], which can help to avoid apoptosis induced by estrogen deficiency in epithelial cells in the salivary gland [70]. Cathepsins are lysosomal cysteine proteases, and their aberrant expression is related to the malignancy of tumors, and thus, the inhibition of cathepsins by *CSTA* expression has been considered to be a target for cancer treatment [71]. Down-regulation of *CSTA* expression after the treatment of E_2_ in ERα-positive breast cancer cells were reported [71, 72]. *CTSD* is an ERG and has been used as a marker of estrogen response [12]. The inhibition of CTSD activity is one of the targets for cancer treatment by controlling the expression of *CSTA* [71]. *CTSD* is also considered to be a target in antibody-based therapy for the treatment of triple-negative breast cancer due to its correlation with poor prognosis of breast cancer and providing a tumor-specific extracellular target [73, 74]. *DOK7* is associated with congenital myasthenic syndromes, a group of neuromuscular disorders caused by impaired neuromuscular transmission, and encodes an adaptor protein which mediates tyrosine kinase signaling to form the neuromuscular synapse [75, 76]. *DOK7* is also associated with malignancy of several types of cancer, including breast cancer, where *DOK7* can inhibit cell proliferation, migration, and invasion of breast cancer by mediating signals through the PI3K/PTEN/AKT pathway, and thus, may act as a tumor suppressor gene [77]. *IGSF1* encodes a transmembrane immunoglobulin superfamily glycoprotein highly expressed in the hypothalamus and in pituitary cells, and has roles in thyroid hormone biosynthesis [78], follicle-stimulating hormone biosynthesis [79], and growth hormone secretion [80]. Although its cellular function has not been clarified, *IGSF1* mutations affecting the symptoms and disease pathologies revealed that extracellular ligands are crucial for its function [81]. *IL20* encodes a proinflammatory cytokine involved in the pathogenesis of inflammatory diseases, such as psoriasis, rheumatoid arthritis, and atherosclerosis, by inducing factors such as TNF-α, IL-1β, MMP-1/13, and MCP-1, and plays important roles in regulating angiogenesis, osteoblastogenesis, and osteoclastogenesis [82, 83]. *IL20* was found to be an ERG [84], and its expression at the mRNA and protein levels in breast cancer cells was up-regulated by estrogen treatment [85]. *INSYN1* encodes an inhibitory postsynaptic density protein involved in postsynaptic inhibition by decreasing inhibitory currents and increasing excitability in the hippocampus [86]. *MATN2* encodes matrilin-2, a member of the matrilin subfamily of extracellular matrix proteins containing von Willebrand factor A and epidermal growth factor-like domains [87]. MATN2 is localized downstream of several growth factor receptors, such as IGF-1R, GPER, and TGFβ1, and involved in tumor progression by stimulating the growth, chemotaxis, and migration of cancer cells [88, 89]. *PDLIM3* encodes a protein containing a PDZ and LIM domain, which are used to bind to the actin cytoskeleton, and localizing at the Z-line of mature muscle fibers, and thus, the gene is likely involved in the development and maintenance of muscle cells [90, 91]. *SUSD3* encodes a cell surface protein with the sushi domain, a structure with a characteristic sandwich arrangement of β-strands, and has been known as an ERG [92]. The possibility of *SUSD3* as a prognostic and diagnostic marker of breast cancer was investigated in several studies, including a DNA microarray analysis [93], a pathological study [94] and an epidemiological study [95].

Additionally, several genes are considered to be tumor suppressor genes. *CCDC68* is a putative tumor suppressor gene in several types of cancer [96], partly through regulating the cell cycle by controlling the degradation of CDK4 [97]. *FRY* encodes an evolutionary conserved protein with diverse functions, such as those controlling cell growth and morphogenesis through FRY-NDR kinase signaling, in invertebrates and vertebrates [98]. In mammals, FRY suppresses nuclear localization of YAP, a transcriptional coactivator with a role in promoting cell proliferation, and thus, may act as a tumor suppressor [99]. *FRY* is required for mammary gland development and may have a role in the suppression of breast cancer cell growth and proliferation [100].

Moreover, the genes associated with breast cancer are included in the 30 ERGs. The expression of *B4GALT1* has been known to quickly respond to estrogen treatment [6, 101], and the ERα-dependent activation of cell proliferation through membrane B4GALT1 implicated its crucial role in breast cancer development [102]. CYP1A1 is involved in estradiol metabolism by the hydroxylation of estradiol to 2-hydroxyestradiol, which may contribute to the anti-estrogenic effects of aryl hydrocarbon receptor ligands such as TCDD [103]. Due to the connection of cholesterol metabolism to cancer, *FDFT1* has been considered as a cancer prognostic marker and a target for anticancer therapy [104]. The involvement of FDFT1 in breast cancer progression through interaction with PGRMC1, a key protein in lipid metabolism, is considered to be a potential basis for breast cancer treatment [105]. LOXL2 stimulates the proliferation of breast cancer cells and contributes to their oncogenic transformation and pathogenesis, suggesting that the development of LOXL2 inhibitors is an effective therapeutic strategy [106]. *SYNE1* encodes nesprin-1, a member of the spectrin family of structural proteins that links the plasma membrane to the actin cytoskeleton [107]. Since *SYNE1* is located 19 kb downstream of *ESR1* (the ERα gene), its association with estrogen action in ovarian cancer [108], endometrial cancer [109], and endometriosis [110] has been reported. *TMPRSS3* was identified as an ERG by DNA microarray analysis, and its transcription level was increased after treatment with E_2_ in MCF-7 cells with the help of cohesin, a protein complex that may connect distant transcriptional regulatory elements with gene promoters [111]. Furthermore, among 150 breast cancer risk regions identified by genome-wide association studies [112], several ERGs identified in this study (*BARX2*, *CBX6*, *KCNN4*, *MYEOV*, *PDZK1* in 300 ERGs) were included as the targets for cancer drivers, transcription factors, and signaling proteins. We also found that some of the ERGs identified here are included in the genes listed in cancer genomics databases such as The Cancer Genome Atlas (TCGA; portal.gdc.cancer.gov) and International Cancer Genome Consortium (ICGC; dcc.icgc.org) (12 genes found in TCGA and 29 genes in ICGC; see S5 Table).

Thirdly, several ERGs are associated with transcriptional regulation. *BARX2* was identified as a homeodomain transcription factor that binds to the transcriptional regulatory regions in the genes encoding cell adhesion molecules (CAMs) [113] and may have important roles in skeletal muscle development, such as digit chondrogenesis, by controlling the expression of CAMs [114]. The coordination of BARX2 with ER was suggested to regulate the growth, survival, and invasion of breast cancer cells [115]. *EGR3* encodes a transcription factor belonging to a family (the early growth response family) of proteins characterized by conserved zinc finger motifs, which are associated with various roles such as the development of muscle spindles [116], the suppression of excessive immune response [117], and the progression of breast cancer [118]. *EGR3*, known as an ERG [119], has been used for evaluation of estrogenic activity of the constituents in foods and medicines [120]. *GATA4* encodes a transcription factor that binds to the GATA motif as a *cis*-element and transactivates the genes for various cell functions such as the development of heart, liver, lung, and urogenital cells [121, 122]. Due to its suppressive effects against ovarian and colorectal cancer cell proliferation, *GATA4* is considered to act as a potential tumor suppressor gene [123, 124]. *GATA4* is also associated with bone differentiation through the signaling pathways such as the ERα and TGFβ pathways [125, 126]. *SPOCD1* encodes a TFIIS-family transcription factor and has been reported to promote the progression and metastasis of several types of cancer, including bladder and ovarian cancers, and osteosarcoma and glioblastoma, by activating signaling pathways such as the EGFR and VEGFR pathways [127–130]. *ZNF521* encodes a zinc-finger transcription factor that has roles in various biological processes involving stem cells, such as erythroid/myeloid/adipocyte/osteoblast differentiation, neural development, and bone formation, and is also implicated in diseases such as pancreatic, hepatic, gastric, bladder, breast, and ovarian cancers [131]. The role of *ZNF521* in ER-positive breast cancer was indicated to be the expressional regulation of ZNF423, a homolog of ZNF521, which forms a heterodimer with ZNF521, through the transcriptional regulation involving ERGs [132].

Fourthly, the ERGs that are explicitly known for estrogen responsiveness include *B4GALT1*, *CSTA*, *CTSD*, *CYP1A1*, *EGR3*, *IL20*, *PKIB*, *RAPGEFL1*, *SUSD3*, and *TMPRSS3* (for *CTSD* and *EGR3*, see Kiyama et al., 2014 [12]). Additionally, the genes known for the association with estrogen signaling and function (some of which are discussed above) are: *BARX2* (breast cancer), *B4GALT1* (breast cancer), *CTSD* (triple-negative breast cancer), *CYP1A1* (estradiol metabolism), *DOK7* (breast cancer), *EGR3* (breast cancer), *FRY* (breast cancer), *GATA4* (bone differentiation), *IL20* (breast cancer and epigenetic regulation), *LOXL2* (breast cancer), *MATN2* (GPER signaling), *PKIB* (breast cancer), *RAB26* (breast cancer), *RAP1GAP* (GPER signaling), *SUSD3* (breast cancer), *SYNE1* (breast cancer), *TMPRSS3* (breast cancer), and *ZNF521* (breast cancer).

### Functions of ERGs through ER-binding

A genome-wide analysis of ERs and RNA polymerase II binding sites by ChIP-seq analysis has revealed a new paradigm of estrogen-mediated gene expression. For example, ChIP-seq analysis revealed the master transcriptional regulator, which may be the best cancer therapeutic target [133]. The mechanisms identified by ChIP-seq include the transcriptions of the genes with E_2_-regulated promoters and enhancers, miRNAs, enhancer RNAs, long-ncRNAs, and anti-sense RNAs [134]. The cistrome, or the genome-wide binding landscape, identified by ChIP-seq using ERα phosphorylated at serine 118 revealed the involvement of this site in a genome-wide transcriptional regulation, such as that by the transcription factor GRHL2, in estrogen action [135]. Such cistromic studies can be applied to develop breast cancer treatment [136].

About one third of the ERGs contained functional ERα-binding sites near their transcription start sites [137]. ChIP-seq analysis along with functional annotations of the genes in the vicinity of ERα-binding sites using databases, such as the GO, KEGG, and GeneGo databases, revealed that the genes with specific functions, such as biological regulation, cellular processes, and developmental processes, are the targets of ERα [138]. Furthermore, specific regulatory networks consisting of transcription factors, modulators, and targets, such as GRHL2, RUNX1, SRC1/SRC2/SRC3, and ERβ, have been identified as potential ER signaling regulators [135, 139–141]. In our study, the ER-subtype specificity among 29 ERGs was 10 ERα-specific, 2 ERβ-specific, and 14 ERα/β-specific genes, along with 3 genes classified as other types (Table 2). Thus, more than 80% of the identified ERGs are ERα-dependent. Interestingly, the ERα non-genomic network is more reliable than the genomic network [141], and thus, may be predominant in MCF-7 cells.

Three ERGs identified here (*ZNF521*, *IGSF1*, and *CRISP3*) were found to be ERα/β-independent, suggesting that they are involved in other signaling pathways. The expression of *ZNF521* is known to potentially be controlled by *ZNF423*, an ERα-dependent ERG [132], and thus, its regulation may be indirect, as observed here. On the other hand, the expressions of *IGSF1* and *CRISP3* are known to be androgen receptor (AR)-dependent [142] and characteristic in prostate cancer cells [143]. The expression and transcriptional regulation of the AR in breast cancer cells, including MCF-7 cells, has been known [144, 145], and the crosstalk between ERα and the AR in breast cancer progression [146, 147] is a likely mechanism of transcriptional regulation of these two genes.

Based on ChIP-seq data, a total of 80,000 potential ERα-binding sites were classified into four groups based on the variations of the presence or absence of both estrogen-responsive elements and the treatment with E_2_ [148], which indicates that some ERα-binding sites are not E_2_-dependent. Evidently, ERα is present prior to ligand activation in the majority of the binding sites [148]. An independent study also revealed the presence of estrogen-independent ER activity such as enhancer activity and the associated activity of histone modifications and enhancer RNA transcription [149]. Additionally, there are differences in ChIP-seq data for ERα-binding sites detected after the treatment with different ligands, E_2_, tamoxifen, and ICI 182,780, suggesting the mechanistic differences of ligand actions [150]. Thus, the ERα/β-independent ERGs may represent the types of transcriptional regulation with more complex mechanisms of estrogen action.

## Conclusions

In this paper, we identified the ERGs that show statistical stability based on the CV values. We evaluated their usefulness as markers for estrogenic activity by examining the stability of the data when compared with correlation analysis, RT-PCR, and functional association with estrogen action through database searches. The ERGs identified here have been known for (1) the enzymes, such as metabolic enzymes, proteases, and protein kinases, (2) the genes with specific cell functions, such as cell-signaling mediators, tumor-suppressors, and roles in breast cancer, (3) the association with transcriptional regulation, and (4) estrogen-responsiveness. The top 30 ERGs were further classified as ERα-binding, ERβ-binding, ERα/β-binding, or ER-independent types based on ChIP-seq data. Therefore, the ERGs identified here represent various cell functions and cell signaling pathways, including estrogen signaling, and thus, may be useful for evaluations of estrogenic activity.

## Supporting information

S1 Table List of the primers for *real-time* RT-PCR analysis for the top 30 ERGs. (XLSX)

S2 Table Correlation analysis of RNA-seq datasets. (DOCX)

S3 Table List of 300 ERGs stably responding to E_2_ and their expression data. A total of 300 ERGs containing 150 up- and 150 down-regulated genes among ERGs stably responding to E_2_.
(XLSX)

S4 Table Functional analysis of ERGs. (A) up-regulated genes in GO analysis; (B) down-regulated genes in GO analysis; (C) up-regulated genes in KEGG analysis; (D) down-regulated genes in KEGG analysis.
(XLSX)

S5 Table List of 30 or 300 ERGs listed in the TGCA and ICGC databases. (DOCX)

S1 Raw images (PDF)
