# Supplementary material for: Novel estrogen-responsive genes (ERGs) for the evaluation of estrogenic activity
Source: PLoS One. 2022 Aug 17;17(8):e0273164. doi: 10.1371/journal.pone.0273164 (PMC9385026; doi:10.1371/journal.pone.0273164)
Supplement: S2 Table — (DOCX) [file pone.0273164.s002.docx]

**S2 Table. Correlation analysis of RNA-seq datasets.**

| Combination | *R* value | *p* value |
| --- | --- | --- |
| 1 vs 2 | 0.95 | 2.07×10^-58^ |
| 1 vs 3 | 0.92 | 6.57×10^-51^ |
| 1 vs 4 | 0.83 | 2.75×10^-32^ |
| 1 vs 5 | 0.90 | 2.64×10^-43^ |
| 1 vs 6 | 0.91 | 5.06×10^-46^ |
| 2 vs 3 | 0.96 | 2.16×10^-63^ |
| 2 vs 4 | 0.84 | 1.17×10^-32^ |
| 2 vs 5 | 0.92 | 5.46×10^-48^ |
| 2 vs 6 | 0.92 | 2.62×10^-48^ |
| 3 vs 4 | 0.81 | 1.70×10^-29^ |
| 3 vs 5 | 0.89 | 7.96×10^-43^ |
| 3 vs 6 | 0.89 | 3.33×10^-41^ |
| 4 vs 5 | 0.86 | 1.92×10^-35^ |
| 4 vs 6 | 0.79 | 6.65×10^-27^ |
| 5 vs 6 | 0.96 | 6.73×10^-65^ |
