# Supplementary material for: Novel estrogen-responsive genes (ERGs) for the evaluation of estrogenic activity
Source: PLoS One. 2022 Aug 17;17(8):e0273164. doi: 10.1371/journal.pone.0273164 (PMC9385026; doi:10.1371/journal.pone.0273164)
Supplement: S5 Table — (DOCX) [file pone.0273164.s005.docx]

**S5 Table. List of 30 or 300 ERGs listed in the TGCA and ICGC databases.**

(1) 300 (or 30; highlighted) ERGs listed in TCGA:

*ZNF521, TP63, COL2A1, SALL4, SRGAP3, MAML2, JAK2, MAF, CDK6, LMO2, JAZF1, LMO1*

(12 genes).

(2) 300 (or 30; highlighted) ERGs listed in ICGC:

*SYNE1, PTPRN2, HMCN1, PCDHA8, ERC2, NAV3, NAV2, TRPC6, CREB5, ZNF521, MAP2, SDK2, PTPRQ, ZNF385B, WSCD1, ACOXL, BMPER, GRIK3, PLCE1, SRGAP3, FRY, NR3C2, TNS3, MAML2, TP63, GSG1L, FRMD3, JAZF1, HMGCLL1, AJAP1, TSHZ3, COLEC12, BRINP2, PPM1E, PGM5, SLIT1, GREB1, PKIB, CACNA1I, SYNPO2, PGR, COL28A1, DAAM2, IGSF1, NR5A2, GRM4, SLC39A10, DEPTOR, CDK6, MATN2, PRICKLE1, GABRA1, ITGB6, PRSS23, NPR3, CD109, BCAS1, FAM184B, MXRA5, CDH26, PNPLA7, GCNT2, SLC2A14, LOXL2, ABCC5, CLMP, SGK3, SNED1, SCNN1B, CXCL12, JAK2, FRK, RIN2, CCNJL, IL1R1, NPNT, ADORA1, FAM188B, MUC3A, DPP4, CLIP4, SPTBN5, DRD2, PPFIA4, MEG3, SYTL5, ACE, LAMB3, EFEMP1, ANKRD33B, SLC7A11, COL2A1, NEURL1, MYZAP, CR2, ACE, RASSF5, SMPD3, KL, CSPG4, PCP4, TMEM45A, GATA4, PTPRH, TMPRSS6, LMO3, FHL2, BLNK, RAP1GAP, MYEOV, SHC2, BARX2, ARHGAP36, CELSR2, FMO1, LINC00472, KCNC1, PADI2, PARP10, WNT2B, SPIRE2, CCDC68, SYN1, ELOVL2, CORO2A, MYBL1, DYRK3, NPY1R, NDUFC2-KCTD14, LIPH, SALL4, PADI3, HERC5, P2RX7, SYS1-DBNDD2, PNPLA3, MUC3A, B4GALT1, TMPRSS4, MSH5-SAPCD1, SLITRK6, CELF6, PDZD7, IL19, SLC6A20, CRISP3, CACNG6, CHRD, GDNF, PELI1, FSD1, LMO1, IRF8, TMPRSS3, SPOCD1, JMJD7-PLA2G4B, CCDC162P, HOPX, GSTM2, ACOX2, ACHE, DOK7, ADA, CAV1, FDFT1, NT5E, PLEKHD1, CYP24A1, HSD17B11, PPAN-P2RY11, POTEJ, PLAT, LMO2, INPP5J, FAM84A, SLC37A2, PDLIM3, ERP27, LINC00939, CCDC110, GPR87, HYAL3, CELF6, MAT1A, CEACAM6, OAS1, MAF, PMP22, ZNF488, TGFB3, STAT5A, SMTNL2, DLK1, SENP3, SCARF1, TH, SYT8, PDK4, KCNN4, SUSD3, PDZK1, FOSB, IL17REL, SOX9, IFITM10, DUSP13, HIST1H3D, HSPA6, LINC00898, CSF1, GBP3, RNF207, RAPGEFL1, TUBA3E, SPINK4, SERPINA1, RSPH4A, WISP2, CTSD, C5AR2, CDSN, TMBIM1, JMJD7, TUBA3D, XKRX, RBM24, C15orf59* (INSYN1), VTN, SLC34A3, CSTA, CYBA, HCAR2, BMF, P2RX2, ISG20, ASCL1, TSPAN1, C1QTNF6, CYP1A1, PLLP, B3GNT8, INHBE, APOL4, TINAGL1, BIK, LINC00052, CYP2D6, RAB26, DMRTA1, CHGA, RNF224, CBX6, APOC3, H19, IL24, EGR3, FOLR1, PTGES, HCAR3, SFXN3, C10orf10, CRABP1, GJB3, ADRB1, LRRC37A2, SNAI1, ALDOC, TFF1, MGP, CCDC89, HAR1B, ITGB2-AS1, IL20, SOCS3, LINC01160, CLPSL2, RPL21, GAS1, SNCG, FAM231D, NBPF4, FAM212B-AS1, FAM72D, CYP4F62P, MT1X, PABPC1L2B, SPANXA2, RGPD6, FAM156B* (289 genes).
