# Supplementary material for: Novel estrogen-responsive genes (ERGs) for the evaluation of estrogenic activity
Source: PLoS One. 2022 Aug 17;17(8):e0273164. doi: 10.1371/journal.pone.0273164 (PMC9385026; doi:10.1371/journal.pone.0273164)

Fig. 1C. E2 P-Erk

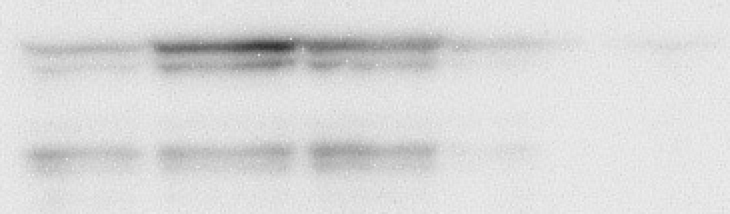

Fig. 1C. E2 T-Erk

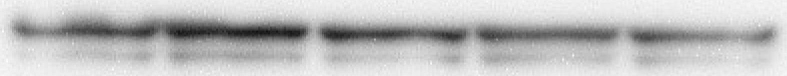

Fig. 1C. E2 P-Akt

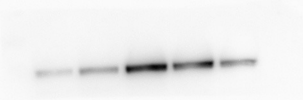

Fig. 1C. E2 T-Akt

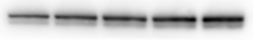

Fig. 1C. E2+ICI P-Erk

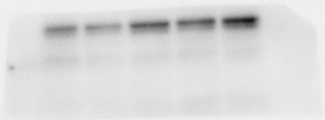

Fig. 1C. E2+ICI T-Erk

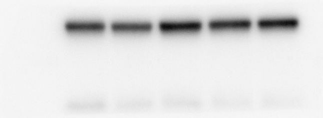

Fig. 1C. E2+ICI P-Akt

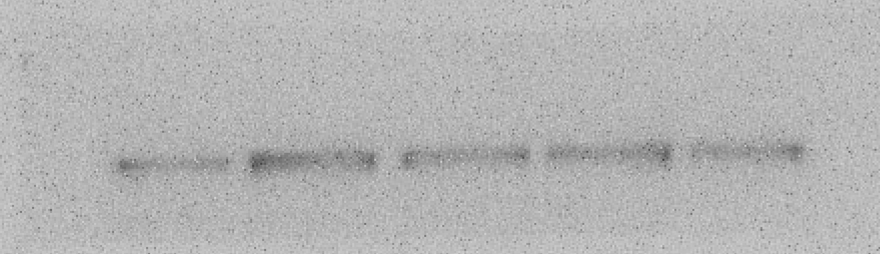

Fig. 1C. E2+ICI T-Akt

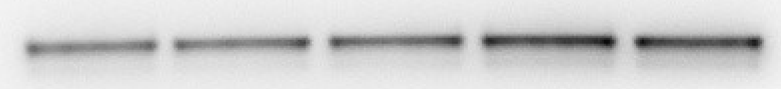

Supplement: S1 Raw images — (PDF) [file pone.0273164.s006.pdf]
